# Beyond Weight Loss and Glycemic Control: Molecular Mechanisms and Hypothesis-Generating Therapeutic Potential of Incretin-Based Therapies Against Peritoneal Fibrosis in Peritoneal Dialysis

**DOI:** 10.3390/ph19101502

**Published:** 2026-09-22

**Authors:** Yi-Chen Li, Jui-Ting Hsu, Chung-Ming Fu, Ting-Chun Liu, Yueh-Ting Lee, Po-Jung Wu, Lung-Chih Li, Terry Ting-Yu Chiou, Chien-Hua Chiu, Wei-Hung Kuo, Chiang-Chi Huang, Kai-Fan Tsai, Tsuen-Wei Hsu, Wen-Chin Lee, Chien-Te Lee, Chih-Chao Yang

**Affiliations:** 1 Clinical Medicine Research Center, National Cheng Kung University Hospital, College of Medicine, National Cheng Kung University, Tainan 701401, Taiwan; n152328@mail.hosp.ncku.edu.tw; 2 Division of Nephrology, Department of Internal Medicine, Kaohsiung Chang Gung Memorial Hospital, Chang Gung University College of Medicine, Kaohsiung 833253, Taiwan; verybiggest80@gmail.com (J.-T.H.); bugfu@cgmh.org.tw (C.-M.F.); tequiero0127@gmail.com (T.-C.L.); yuai@cgmh.org.tw (Y.-T.L.); b9302057@cgmh.org.tw (P.-J.W.); r5239@cgmh.org.tw (L.-C.L.); cyang@cgmh.org.tw (T.T.-Y.C.); e22209@cgmh.org.tw (C.-H.C.); b8701144@cgmh.org.tw (W.-H.K.); a9259@cgmh.org.tw (C.-C.H.); b9302095@cgmh.org.tw (K.-F.T.); scwei@cgmh.org.tw (T.-W.H.); leewc@cgmh.org.tw (W.-C.L.); 3 Division of Nephrology, Kaohsiung Municipal Fong-Shan Hospital, Kaohsiung 83062, Taiwan; 4 Division of Nephrology, Kaohsiung Municipal Ta-Tung Hospital, Kaohsiung 80145, Taiwan

**Keywords:** peritoneal dialysis, peritoneal fibrosis, incretin-based therapy, GLP-1 receptor agonist, dulaglutide, DPP-4, mesothelial-to-mesenchymal transition, oxidative stress, inflammation, TGF-β

## Abstract

**Background:** Peritoneal dialysis (PD) is an established kidney replacement therapy, but long-term exposure to bioincompatible dialysate, uremic toxins, recurrent peritonitis, glucose degradation products, oxidative stress, and sterile inflammation progressively remodels the peritoneal membrane and contributes to peritoneal fibrosis (PF). This review summarizes the molecular basis of PF in PD and evaluates the therapeutic rationale for incretin-based interventions beyond weight loss and glycemic control. **Methods:** This narrative review synthesizes mechanistic, translational, and clinically relevant literature on PD-associated PF, with emphasis on DPP-4 biology, GLP-1 receptor agonism, inflammatory and oxidative signaling, angiogenesis, mesothelial-to-mesenchymal transition, glycemic management in PD, and selected author-conducted translational studies interpreted alongside independent evidence. **Results:** Available evidence supports a model in which DPP-4 activation, altered GLP-1/GLP-1R signaling, TGF-β/Smad3-driven mesothelial transition, NF-κB/MyD88 inflammation, ROS-mediated oxidative stress, angiogenesis, apoptosis, and cytoskeletal remodeling converge to impair peritoneal membrane integrity. DPP-4 inhibition, exendin-4, and dulaglutide attenuated these pathways in experimental models, while GLP-1 receptor agonists have established metabolic benefits in appropriate diabetic and obese populations. **Conclusions:** Direct clinical evidence that incretin-based therapies prevent or treat peritoneal fibrosis in humans undergoing PD is currently lacking. Therefore, peritoneal membrane protection, reduced ultrafiltration failure, and improved technique survival should be regarded as investigational, hypothesis-generating possibilities that require prospective clinical validation.

## 1. Introduction

Peritoneal dialysis (PD) relies on the peritoneal membrane as a living semipermeable interface for solute exchange and ultrafiltration (UF). With prolonged therapy, however, the membrane is exposed to nonphysiological dialysate composition, high glucose concentrations, glucose degradation products (GDPs), advanced glycation end products (AGEs), acidic pH, uremic toxins, and episodes of infectious or sterile peritonitis [1,2,3,4,5]. These stressors initiate a maladaptive repair program involving mesothelial cell injury, inflammatory cell infiltration, fibroblast activation, vascular remodeling, and extracellular matrix (ECM) deposition [6,7]. The clinical consequence is progressive membrane dysfunction, reduced UF capacity, and eventual technique failure [8,9,10]. The interplay of these stressors, molecular mechanisms, and downstream clinical consequences is summarized schematically in Figure 1.

Incretin-based therapies have transformed the management of type 2 diabetes and obesity, but their biological actions extend beyond both body weight reduction and glycemic control [11,12,13,14]. Glucagon-like peptide-1 receptor agonists (GLP-1RAs) modulate inflammation, oxidative stress, endothelial function, mitochondrial homeostasis, natriuresis, appetite regulation, tissue remodeling, and cardiometabolic risk [15,16,17,18]. The present review focuses on whether these established metabolic and cardiorenal effects intersect with PD-specific injury pathways; evidence from MASLD/MASH [19,20] and neurocognitive research [21,22] is used only as limited biological context and is not treated as evidence of peritoneal benefit. Peritoneal protection remains a biologically plausible but unproven extension because PD-associated injury is driven by metabolic stress, oxidative injury, inflammation, vascular remodeling, and fibrosis [23,24,25,26].

## 2. Methods

This article was designed as a narrative review [27]. PubMed/MEDLINE, Embase, Web of Science, Scopus, and Google Scholar were searched from database inception to 30 June 2026. Searches were restricted primarily to English-language, peer-reviewed literature. Eligible sources included mechanistic studies, preclinical experiments, observational studies, clinical reports, guidelines, and relevant reviews addressing PD-associated PF, peritoneal membrane remodeling, mesothelial-to-mesenchymal transition (MMT), inflammatory and oxidative signaling, angiogenesis, lymphangiogenesis, DPP-4 biology, GLP-1 receptor agonists, dual incretin agonists, and preclinical models of peritoneal injury.

Search terms included combinations of “peritoneal dialysis,” “peritoneal fibrosis,” “ultrafiltration failure,” “mesothelial-to-mesenchymal transition,” “TGF-β,” “Smad,” “oxidative stress,” “DPP-4,” “GLP-1 receptor agonist,” “incretin therapy,” “dulaglutide,” “chronic kidney disease,” and “renal fibrosis.” Articles were excluded when they were non-peer-reviewed, unrelated to peritoneal membrane biology or incretin-based therapy, lacked sufficient methodological detail, or addressed only unrelated metabolic outcomes without mechanistic or clinical relevance to PD. Study selection was thematic rather than systematic and was intended to identify biologically convergent pathways, translational gaps, and clinically testable hypotheses. Recent evidence was prioritized for clinical practice, pharmacology, and safety, whereas foundational studies were retained when they established core peritoneal pathobiology or model systems. Independent evidence was prioritized when available, and author-conducted studies were appraised using the same criteria. Formal PRISMA screening, risk-of-bias scoring, and meta-analysis were not performed.

Two author-conducted translational studies were incorporated because they directly addressed the DPP-4/GLP-1 axis in PD-related peritoneal injury; however, they were interpreted cautiously and as hypothesis-generating rather than definitive clinical evidence. The first combined a 17-year Taiwan National Health Insurance Research Database analysis of diabetic patients with kidney failure receiving PD with in vitro mesothelial cell experiments and chlorhexidine gluconate (CG)-induced rat PF models to evaluate DPP-4 as a pathogenic and therapeutic target [28]. The second used Met-5A mesothelial cells, TGF-β/DPP-4 double silencing, p-cresol, CG, lipopolysaccharide (LPS) stimulation, CKD-associated PF models, and LPS-induced peritonitis models to assess dulaglutide as a GLP-1RA-based intervention [29].

## 3. Pathobiology of Peritoneal Fibrosis in Peritoneal Dialysis

PF develops through overlapping cellular and molecular events rather than a single linear pathway [30,31]. Chronic dialysate exposure and uremia impair mesothelial cell integrity, while recurrent inflammation promotes cytokine release and leukocyte recruitment [32,33]. Transforming growth factor-β (TGF-β) is a central profibrotic mediator that activates Smad-dependent transcription, induces myofibroblast differentiation, and increases expression of collagen I, fibronectin, α-smooth muscle actin (α-SMA), and other ECM components [34,35,36]. In parallel, non-Smad pathways such as mitogen-activated protein kinase (MAPK), phosphoinositide 3-kinase (PI3K)/Akt, Wnt/β-catenin, and nuclear factor-κB (NF-κB) amplify inflammatory and fibrotic responses [37,38,39].

MMT is a hallmark of peritoneal remodeling [40,41]. Injured mesothelial cells lose epithelial features, acquire migratory and contractile properties, and contribute to submesothelial thickening [42]. Oxidative stress generated by reactive oxygen species (ROS), mitochondrial dysfunction, NADPH oxidase (NOX) activation, and AGEs reinforces this transition [43,44]. Angiogenesis and lymphangiogenesis further worsen UF by increasing effective vascular surface area and solute transport [45,46], whereas persistent immune activation sustains a fibrogenic microenvironment [47].

One author-conducted study provides mechanistic evidence linking dialysis-related metabolic stress with peritoneal injury, but its findings require independent replication and should not be interpreted as proof of clinical membrane protection. High-glucose exposure upregulated DPP-4 in Met-5A mesothelial cells, reduced GLP-1 expression, activated phosphorylated Smad3 and NF-κB signaling, increased NOX-1 and NOX-2, suppressed antioxidant defenses such as SOD1 and NQO1, and promoted a mesenchymal phenotype [28]. In vivo, chlorhexidine gluconate increased peritoneal DPP-4 expression, submesothelial thickening, collagen deposition, angiogenesis, leukocyte infiltration, oxidative stress, and transport dysfunction [28]. Serum and peritoneal-fluid DPP-4 activity correlated with PET abnormalities, but these correlations require prospective validation before DPP-4 can be considered a clinical biomarker. Together, these findings support a DPP-4/GLP-1 axis-centered model of dialysis-related peritoneal injury, as summarized in Figure 2.

## 4. Incretin-Based Therapies: Biology Beyond Weight Loss and Glycemic Control

Incretin-based therapies include GLP-1RAs, dual incretin agonists, and DPP-4 inhibitors [48,49]. GLP-1RAs enhance glucose-dependent insulin secretion, suppress glucagon release and slow gastric emptying, and reduce appetite [50]. However, experimental and clinical studies increasingly support additional cardiorenal actions, including attenuation of inflammation, oxidative stress, endothelial dysfunction, albuminuria, and progressive renal decline [51,52]. These effects are mediated through both direct receptor-dependent pathways and indirect improvements in metabolic, hemodynamic, and inflammatory burden [53].

The pleiotropic scope of GLP-1RA therapy has also been discussed in MASLD/MASH [19,20] and neurodegenerative research [21,22]; however, these areas are mentioned here only to illustrate overlapping metabolic, inflammatory, vascular, mitochondrial, and cellular stress pathways. Definitive clinical benefits outside approved metabolic and cardiorenal indications remain disease-specific and should not be extrapolated to PD-associated PF. Peritoneal protection is therefore best viewed as a biologically plausible but unproven translational hypothesis because the peritoneal membrane is exposed to chronic glucose, uremic, inflammatory, oxidative, angiogenic, and profibrotic stress during PD [1,2,3,4,5,28,29].

For PD, the most relevant nonmetabolic properties include suppression of NF-κB signaling, reduction in inflammatory mediators, enhancement of antioxidant defenses, preservation of mitochondrial function, and inhibition of TGF-β-driven ECM accumulation. However, evidence for functional GLP-1R expression and receptor-dependent signaling in relevant human peritoneal mesothelial, fibroblast, endothelial, and immune-cell populations remains limited. Apparent peritoneal effects may therefore arise from direct receptor signaling, indirect metabolic improvement, altered hemodynamics, systemic anti-inflammatory actions, weight loss, or combinations of these mechanisms. Findings from DPP-4 inhibition, genetic DPP-4 deficiency, exendin-4, or dulaglutide are agent- and model-specific and should not be generalized automatically to all GLP-1RAs or dual/multi-agonists.

The same study further showed that incretin-based interventions could attenuate these injury pathways in experimental systems [28]. Genetic DPP-4 deficiency, sitagliptin, and exendin-4 comparably attenuated chlorhexidine-induced peritoneal thickening, collagen I accumulation, TGF-β/Smad3 activation, NF-κB/MyD88 signaling, NOX-1 expression, inflammatory cell infiltration, angiogenesis, and abnormal peritoneal transport [28]. Clinically, the nationwide cohort analysis found that diabetic patients receiving PD and DPP-4 inhibitors had a lower transition rate to hemodialysis than matched nonusers. This association does not establish preservation of the peritoneal membrane or prevention of fibrosis, because PD-to-HD transition may reflect infection, inadequate dialysis, catheter or mechanical problems, psychosocial factors, patient preference, transplantation planning, cardiovascular instability, or other causes. Residual confounding, treatment-selection bias, exposure misclassification, and absence of direct histologic or longitudinal membrane measures further limit causal interpretation. The candidate pathways and structured evidence levels are summarized in Table 1 and Table 2.

Diabetes management in peritoneal dialysis is uniquely challenging because glucose-based dialysate provides a continuous and variable exogenous glucose load [54]. Hypertonic exchanges may increase systemic glucose absorption, worsen postprandial and nocturnal hyperglycemia, increase glycemic variability, promote dyslipidemia and weight gain, and complicate insulin dose adjustment [54]. At the same time, kidney failure reduces insulin clearance and impairs counter-regulatory responses, increasing vulnerability to hypoglycemia when insulin or insulin secretagogues are intensified [54]. Therefore, an ideal antidiabetic strategy in peritoneal dialysis should improve glycemic control while minimizing hypoglycemia, excessive weight gain, and treatment burden [54].

DPP-4 inhibitors are attractive in this setting because they enhance endogenous incretin activity in a glucose-dependent manner. Evidence for glucose lowering and low intrinsic hypoglycemia risk is established mainly in type 2 diabetes and broader CKD populations [55,56]; PD-specific clinical evidence remains limited. Several DPP-4 inhibitors require renal dose adjustment, whereas linagliptin is less dependent on renal excretion. In the author group’s nationwide PD cohort, DPP-4 inhibitor use was associated with lower PD-to-HD transition; this observation is exploratory, is not a direct measure of PF or membrane preservation, and requires prospective independent validation.

GLP-1 receptor agonists stimulate glucose-dependent insulin secretion, suppress inappropriate glucagon secretion, slow gastric emptying, reduce appetite, and improve postprandial glucose excursions [11,12]. Cardiovascular, kidney, weight, and insulin-sparing benefits are supported predominantly by trials in type 2 diabetes, obesity, or non-dialysis CKD; evidence in kidney failure or dialysis is smaller, and direct PD-specific evidence is sparse. Accordingly, potential use in PD should be individualized and should not imply demonstrated peritoneal membrane benefit.

The low intrinsic hypoglycemia risk of GLP-1RAs may be useful when insulin requirements are variable, but concomitant insulin often requires adjustment [54,57,58]. Weight loss is not uniformly beneficial in PD. Patients with obesity who seek cardiometabolic improvement or transplant eligibility differ substantially from older, frail, or low-body-mass patients who are vulnerable to sarcopenia, poor oral intake, peritoneal protein losses, and protein-energy wasting. Nausea, vomiting, diarrhea, early satiety, and delayed gastric emptying may further reduce energy and protein intake or contribute to volume depletion. Baseline phenotype, dietary intake, weight trajectory, muscle mass or functional status, and residual kidney function should therefore guide treatment and follow-up.

Despite these advantages, glycemic targets and drug selection must remain individualized. HbA1c may be less reliable in dialysis because of anemia, altered red blood cell survival, erythropoiesis-stimulating agents, and transfusion exposure. Continuous glucose monitoring (CGM) or structured capillary glucose profiles may better capture dialysate-related glycemic variability, nocturnal hyperglycemia, and hypoglycemia risk. Dialysis-specific safety monitoring is essential when GLP-1RAs are used, including nausea, vomiting, diarrhea, delayed gastric emptying, appetite suppression, reduced caloric or protein intake, protein-energy wasting, sarcopenia risk, volume depletion, residual kidney function, and the need to reduce insulin doses when glycemic control improves. DPP-4 inhibitors also differ pharmacokinetically: linagliptin is minimally renally cleared, whereas sitagliptin, saxagliptin, alogliptin, and vildagliptin generally require kidney-function-based dose adjustment. Thus, incretin-based therapy in PD should be framed as a metabolic and cardiorenal strategy with possible but unproven peritoneal membrane benefits.

## 5. Molecular Rationale for Targeting Peritoneal Fibrosis with Incretin-Based Therapies

The mechanistic rationale can be summarized in four intersecting, experimentally supported pathways rather than interpreted as clinical efficacy: attenuation of TGF-β/Smad3 signaling and matrix deposition; reduction in NF-κB/MyD88-driven inflammation; modulation of NOX-related oxidative stress and antioxidant defenses; and preservation of mitochondrial viability [28,29]. These pathways overlap with the general biology of PF described above and are not repeated here as evidence of patient benefit.

In one author-conducted preclinical study, dulaglutide reduced oxidative, inflammatory, DPP-4, and TGF-β/Smad3 signals in Met-5A cells exposed to p-cresol or LPS and improved cell viability and mitochondrial oxidative-stress measures [29]. TGF-β/DPP-4 double silencing supported involvement of these pathways. Because these experiments used an immortalized cell line and selected stimuli, they establish mechanistic plausibility rather than efficacy in human PD.

In CKD rats exposed to chlorhexidine, dulaglutide reduced peritoneal thickening, fibrosis, vascular remodeling, inflammatory-cell infiltration, and selected MMT, oxidative, inflammatory, angiogenic, and DPP-4-related markers while increasing GLP-1R, Nrf2, and NQO-1 [29]. These findings are agent-specific and preclinical; they have not been independently replicated in a chronic human PD exposure model.

These models have important translational limitations. Chlorhexidine-induced fibrosis produces severe chemical serosal injury and does not reproduce the cumulative glucose exposure, glucose-degradation products, recurrent infection, uremia, mechanical forces, and heterogeneous time course of chronic human PD. Likewise, LPS-induced peritonitis is a standardized endotoxin model rather than clinical PD-associated infectious peritonitis, which varies by organism, inoculum, antimicrobial treatment, catheter factors, and host immunity. Accordingly, improvement in these models cannot be assumed to predict membrane preservation or peritonitis recovery in patients.

In an LPS-induced peritonitis model, dulaglutide reduced circulating and abdominal-fluid DPP-4, decreased experimental permeability, and lowered selected inflammatory-cell populations [29]. These findings support an acute endotoxin-response hypothesis only and should not be described as evidence of clinical PD-associated peritonitis recovery.

## 6. Therapeutic Potential and Clinical Translation

The therapeutic appeal of incretin-based approaches in PD lies in their capacity to target multiple pathogenic domains simultaneously. A successful antifibrotic intervention would ideally reduce inflammatory signaling, oxidative injury, angiogenesis, mesothelial transition, and ECM accumulation without compromising host defense or dialysis adequacy [1,2,3,4,5,28,29]. GLP-1RAs may be especially relevant for patients with DM, obesity, cardiovascular risk, or CKD, in whom these agents already have established metabolic and cardiorenal indications [51,52].

Nonetheless, several translational questions remain unresolved. Direct clinical evidence in PD-associated fibrosis is limited [3,4,5]. The optimal agent, dose, treatment timing, duration, and patient phenotype are unknown. It is also unclear whether benefit would be greatest as primary prevention early in PD, as rescue therapy in patients with high transport status, or as adjunctive therapy following peritonitis. Safety considerations include gastrointestinal intolerance, nutritional status, residual kidney function, fluid balance, and the pharmacokinetic implications of advanced kidney disease [54]. Carefully designed clinical trials using peritoneal equilibration testing, UF measures, effluent biomarkers, imaging, and, when appropriate, histologic endpoints will be required.

The two author-conducted studies are considered alongside, rather than above, independent evidence. They provide complementary observational and preclinical signals, but both lack prospective human membrane endpoints and independent replication. Additional limitations include reliance on administrative coding and a non-randomized exposure definition in the cohort analysis, use of an immortalized mesothelial cell line, severe chemical or endotoxin injury models, short experimental follow-up, and uncertain generalizability across incretin agents. Their principal value is to generate testable hypotheses and exploratory biomarkers, not to establish clinical efficacy.

## 7. Discussion and Clinical Implications

For clinicians, incretin-based therapy in PD should currently be considered only within approved metabolic or cardiorenal indications and individualized patient goals. Established evidence is strongest outside PD; PD observations are limited; and membrane protection remains an experimental hypothesis. Conventional membrane-preserving care continues to include individualized prescriptions, avoidance of unnecessary glucose exposure, icodextrin when appropriate, prevention and prompt treatment of peritonitis, and regular assessment of peritoneal transport.

Evidence from MASLD/MASH [19,20] and neurocognitive research [21,22] provides only limited pleiotropic context and does not support peritoneal efficacy. PD-specific priorities are cumulative dialysate glucose exposure, glycemic variability, nutritional status, gastrointestinal tolerability, pharmacokinetic constraints in kidney failure, residual kidney function, and the absence of validated clinical membrane outcomes. Consequently, potential peritoneal protection should be tested independently using functional and biomarker-rich PD studies rather than inferred from benefits in other organ systems.

From a diabetes care perspective, incretin-based therapies may be particularly useful for peritoneal dialysis patients who experience hyperglycemia after high-glucose exchanges, excessive glycemic variability, weight gain, or escalating insulin requirements. Their glucose-dependent mechanism may reduce hypoglycemia risk compared with therapies that stimulate insulin secretion independently of ambient glucose [57,58]. DPP-4 inhibitors may be considered when oral therapy, simplicity, and tolerability are priorities, whereas GLP-1 receptor agonists may be favored when weight control, postprandial hyperglycemia, cardiovascular risk reduction, and insulin-sparing effects are important clinical goals. In patients with overweight or obesity, GLP-1RAs may also help counter PD-related weight gain and support transplant candidacy or cardiometabolic risk reduction, although appetite suppression and gastrointestinal adverse effects require careful nutritional monitoring in dialysis populations [59,60].

In current practice, however, incretin-based therapy should not be regarded as an established antifibrotic treatment for PD-associated membrane injury. Its use should follow approved metabolic and cardiorenal indications, while potential membrane benefits remain investigational. Future clinical studies should consider enrolling patients at high risk for membrane deterioration, including individuals with DM, obesity, high peritoneal solute transfer rate, recurrent peritonitis, persistent inflammatory effluent profiles, or early decline in UF capacity. Relevant outcomes should include changes in PET parameters, net UF, dialysate-to-plasma creatinine ratio, sodium sieving, effluent cytokines, DPP-4 activity, matrix remodeling biomarkers, hospitalization, technique survival, nutrition, gastrointestinal tolerability, and patient-reported quality of life.

Future pilot studies should enroll clearly defined PD phenotypes and combine clinical outcomes with mechanistic readouts. Candidate biomarkers, including serum or effluent DPP-4 activity and MMT-related markers, remain exploratory and require assay standardization, prospective validation, and independent replication before use for selection or treatment monitoring. The proposed clinical and mechanistic endpoints for this translational framework are summarized in Figure 3.

## 8. Future Directions

**Mechanistic validation:** Define GLP-1R and DPP-4 expression and signaling in human mesothelial cells, fibroblasts, macrophages, endothelial cells, and peritoneal tissue from patients undergoing peritoneal dialysis.

**Biomarker discovery:** Prospectively and independently validate serum and effluent DPP-4 activity, GLP-1/GLP-1R signaling, NOX-1/NOX-2, Nrf2/NQO-1, NF-κB/MyD88, TGF-β/Smad3, MMP-9, and MMT markers before they are used to predict transport decline or treatment response.

**Comparative pharmacology:** Compare DPP-4 inhibitors, short-acting and long-acting GLP-1RAs, and future dual or multi-agonists in standardized models, while avoiding class-wide inference from results obtained with a single compound.

**Clinical trial design:** Develop biomarker-rich pilot studies evaluating membrane transport status, ultrafiltration capacity, peritonitis recovery, effluent inflammatory phenotypes, permeability, glycemic control, continuous glucose monitoring metrics, insulin requirement, hypoglycemia frequency, body weight, quality of life, and technique survival.

**Patient stratification:** Determine whether diabetes status, obesity, DPP-4 activity, GLP-1R expression, baseline inflammation, high transport phenotype, glucose exposure, uremic toxin burden, prior peritonitis, glycemic variability, insulin requirement, or hypoglycemia risk modifies therapeutic response.

## 9. Conclusions

PF remains a major barrier to durable PD. Available support for incretin-based membrane protection is mechanistic, agent-specific preclinical, and observational; clinical antifibrotic efficacy has not been demonstrated.

Incretin-based agents should therefore be used in PD only for established indications and individualized metabolic goals. Prospective PD trials are required before these therapies can be considered interventions for membrane preservation, ultrafiltration failure, peritonitis-related injury, or technique survival.

## Figures and Tables

**Figure 1:**
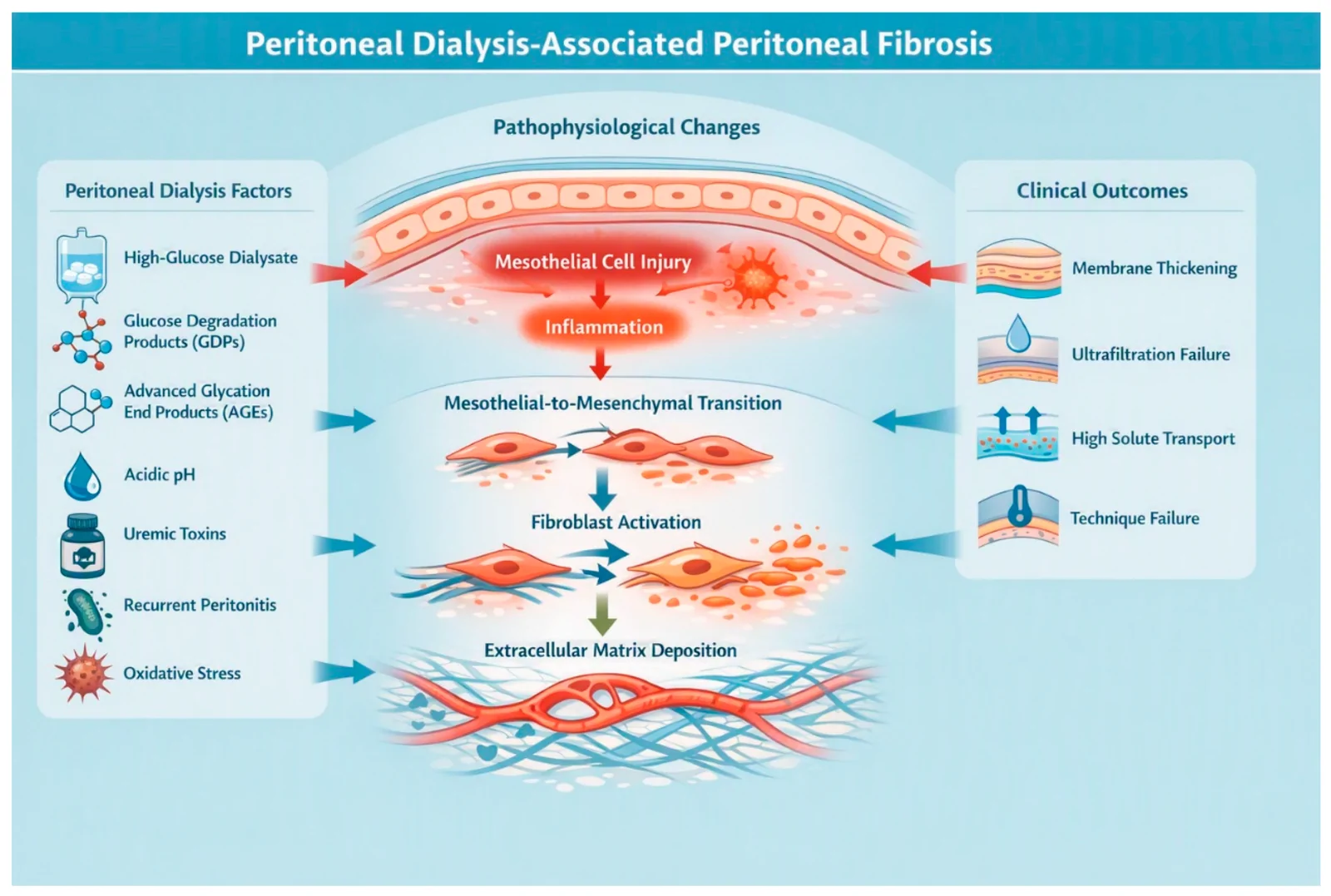
Integrated overview of peritoneal dialysis-associated peritoneal fibrosis. Chronic exposure to bioincompatible dialysate, uremic toxins, glucose degradation products, recurrent peritonitis, oxidative stress, and inflammatory stimuli drives mesothelial injury, mesothelial-to-mesenchymal transition, fibroblast activation, angiogenesis, extracellular matrix deposition, membrane thickening, ultrafiltration failure, and eventual technique failure. **Red arrows:** Indicate acute injury and inflammatory pathways, showing how high-glucose dialysate triggers mesothelial cell injury and acute inflammation, ultimately leading to membrane thickening. **Blue arrows:** Represent contributing chronic peritoneal dialysis factors (such as glucose degradation products, advanced glycation end products, acidic pH, uremic toxins, recurrent peritonitis, and oxidative stress) as well as downstream clinical outcomes (such as ultrafiltration failure, high solute transport, and technique failure).

**Figure 2:**
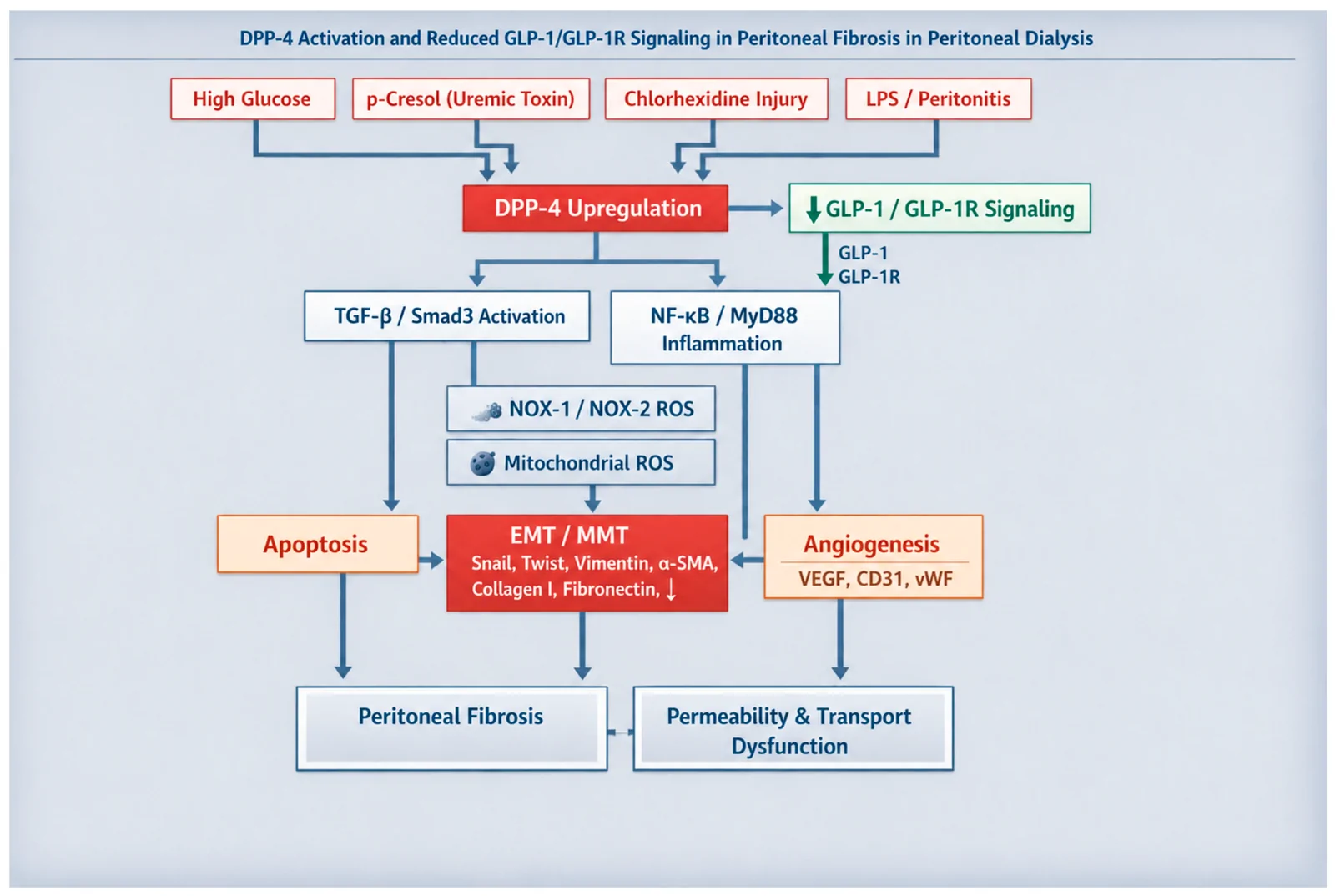
Proposed DPP-4/GLP-1 axis-centered mechanism linking dialysis-related stress to peritoneal fibrosis. High-glucose exposure, uremic toxins, chlorhexidine injury, and endotoxin-mediated inflammation may increase DPP-4 activity and suppress protective GLP-1/GLP-1R signaling, thereby promoting TGF-β/Smad3 activation, NF-κB/MyD88 inflammation, NOX-mediated oxidative stress, mitochondrial injury, apoptosis, angiogenesis, mesothelial-to-mesenchymal transition, extracellular matrix accumulation, and transport dysfunction. Abbreviations: DPP-4, dipeptidyl peptidase-4; GLP-1, glucagon-like peptide-1; GLP-1R, glucagon-like peptide-1 receptor; TGF-β, transforming growth factor-beta; Smad3, suppressor of mothers against decapentaplegic homolog 3; NF-κB, nuclear factor kappa B; MyD88, myeloid differentiation primary response 88; NOX-1/2, NADPH oxidase 1/2; NADPH, nicotinamide adenine dinucleotide phosphate; ROS, reactive oxygen species; EMT, epithelial-to-mesenchymal transition; MMT, mesothelial-to-mesenchymal transition; α-SMA, alpha-smooth muscle actin; VEGF, vascular endothelial growth factor; CD31, cluster of differentiation 31 (platelet endothelial cell adhesion molecule-1); vWF, von Willebrand factor; LPS, lipopolysaccharide.

**Figure 3:**
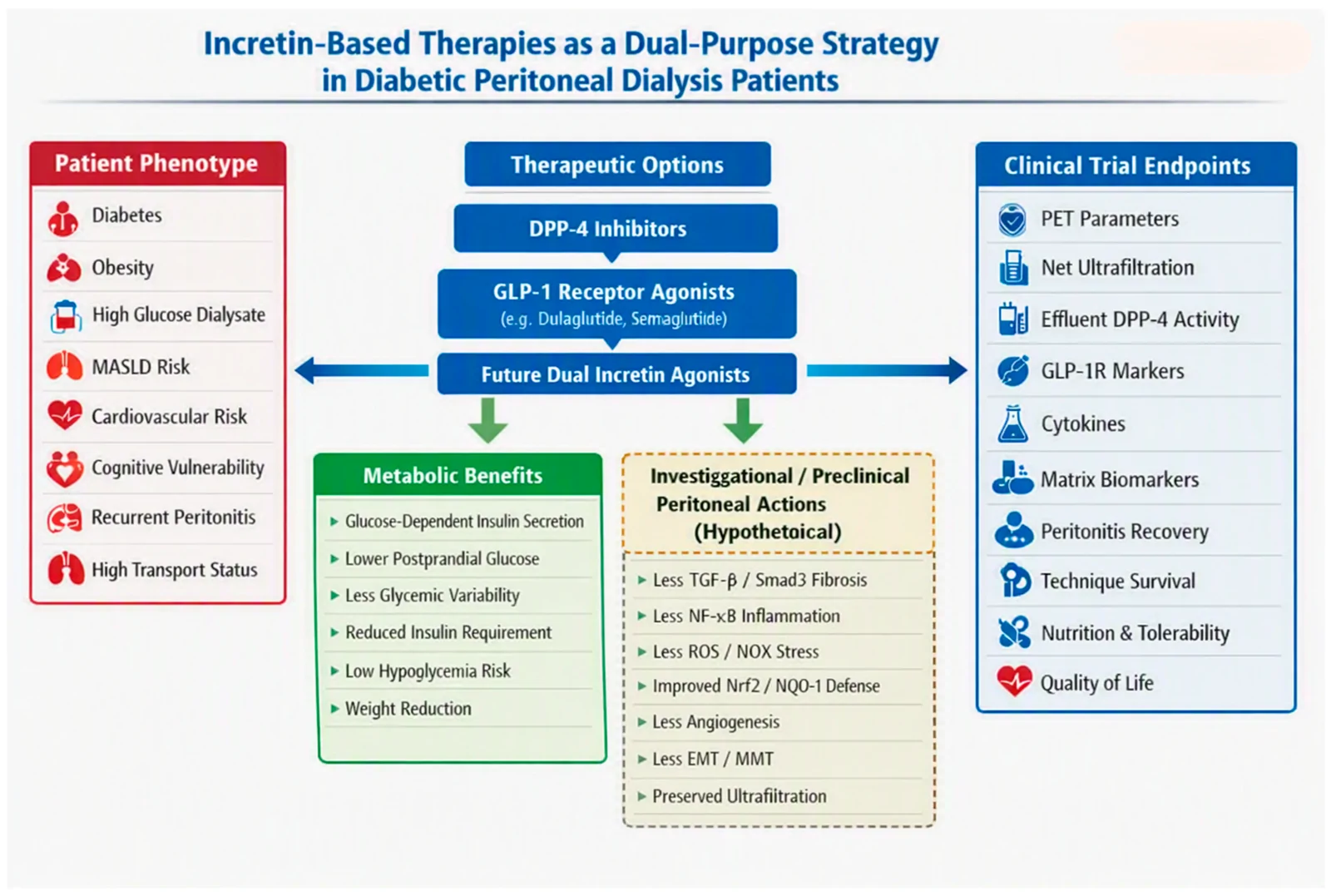
Evidence-stratified framework for future investigation. Established clinical effects are limited to approved metabolic and selected cardiorenal outcomes in appropriate non-PD and broader CKD populations. PD-specific metabolic observations are limited. Peritoneal anti-inflammatory, antifibrotic, permeability, membrane-preservation, and technique-survival effects are preclinical or hypothetical and require prospective trials. Candidate studies should evaluate glycemic metrics, insulin requirement, hypoglycemia, nutritional status, gastrointestinal tolerability, peritoneal transport, ultrafiltration, exploratory effluent biomarkers, quality of life, and technique survival.

**Table 1:** Candidate mechanistic pathways linking peritoneal fibrosis and incretin-based therapy.

Pathway or Process	Role in Peritoneal Fibrosis	Potential Incretin-Related Action	Candidate Biomarkers or Endpoints
TGF-β/Smad signaling	Promotes myofibroblast differentiation and extracellular matrix deposition.	May reduce Smad3 phosphorylation and profibrotic transcription.	TGF-β, phosphorylated Smad3, collagen I, fibronectin, α-SMA.
NF-κB-mediated inflammation	Amplifies cytokine release, immune-cell recruitment, and mesothelial injury.	May suppress inflammatory transcription and cytokine production.	NF-κB, TNF-α, IL-6, MCP-1, effluent leukocyte profile.
Oxidative stress	Drives mitochondrial injury, apoptosis, EMT/MMT, and matrix accumulation.	May reduce NADPH oxidase activity and enhance antioxidant responses.	NOX-1, NOX-2, ROS, Nrf2, NQO1, oxidative stress markers.
DPP-4 activity	May contribute to inflammatory and fibroblast-activating networks.	GLP-1 pathway enhancement or DPP-4 modulation may reduce injury signaling.	DPP-4 expression or activity, GLP-1 receptor expression.
Angiogenesis and lymphangiogenesis	Increase vascular surface area and solute transport, worsening ultrafiltration.	May reduce angiogenic signaling associated with inflammatory injury.	VEGF-related markers, CD31, von Willebrand factor, PSTR, net ultrafiltration.
Mesothelial-to-mesenchymal transition	Contributes to submesothelial thickening and fibrotic remodeling.	May preserve epithelial phenotype and reduce mesenchymal markers.	E-cadherin, N-cadherin, vimentin, Snail, β-catenin, α-SMA.
Dialysate-related glycemic burden	Glucose absorption from PD solutions worsens hyperglycemia, glycemic variability, insulin resistance, dyslipidemia, and weight gain.	DPP-4 inhibitors and GLP-1 receptor agonists may improve glucose-dependent insulin secretion, suppress glucagon, reduce postprandial excursions, and lower insulin requirements with limited intrinsic hypoglycemia risk.	HbA1c, glycated albumin, CGM time-in-range, glucose variability, hypoglycemia frequency, insulin dose, body weight, lipid profile.

**Table 2:** Structured summary of evidence by agent, model, intervention, endpoint, and evidence level.

Agent or Approach	Model or Setting	Intervention or Exposure	Main Endpoints	Evidence Level
DPP-4 inhibition	Nationwide diabetic PD cohort; mesothelial cells; CG-induced rat PF	DPP-4 inhibitor exposure, DPP-4 deficiency, sitagliptin	PD-to-HD transition, DPP-4 activity, MMT markers, oxidative stress, inflammation, angiogenesis, transport dysfunction	Observational plus preclinical; hypothesis-generating
Exendin-4	CG-induced rodent PF model	GLP-1 receptor agonism	Peritoneal thickening, collagen deposition, inflammatory signaling, angiogenesis	Preclinical
Dulaglutide	Met-5A cells; CKD plus CG model; LPS peritonitis model	Long-acting GLP-1RA under uremic toxin, fibrogenic, and endotoxin stress	ROS, mitochondrial injury, apoptosis, NF-κB/MyD88, TGF-β/Smad3, permeability, inflammatory-cell recruitment	Preclinical; no definitive human antifibrotic evidence
Future dual or multi-agonists	PD-associated PF	Not yet established	Peritoneal transport, effluent biomarkers, ultrafiltration, nutrition, tolerability, technique survival	Hypothetical; requires prospective trials

## Data Availability

No new data were created or analyzed in this study. Data sharing is not applicable to this article.

## Abbreviations

The following abbreviations are used in this manuscript:

**Abbreviation**	**Definition**
AGE	advanced glycation end product
CG	chlorhexidine gluconate
CGM	continuous glucose monitoring
CKD	chronic kidney disease
DM	diabetes mellitus
DPP-4	dipeptidyl peptidase-4
ECM	extracellular matrix
EMT	epithelial-to-mesenchymal transition
GDP	glucose degradation product
GLP-1	glucagon-like peptide-1
GLP-1R	glucagon-like peptide-1 receptor
GLP-1RA	glucagon-like peptide-1 receptor agonist
HD	hemodialysis
LPS	lipopolysaccharide
MASLD	metabolic dysfunction-associated steatotic liver disease
MASH	metabolic dysfunction-associated steatohepatitis
MMP-9	matrix metalloproteinase-9
MMT	mesothelial-to-mesenchymal transition
MyD88	myeloid differentiation primary response 88
NF-κB	nuclear factor-κB
NOX	NADPH oxidase
NQO-1	NAD(P)H quinone oxidoreductase 1
Nrf2	nuclear factor erythroid 2-related factor 2
PD	peritoneal dialysis
PET	peritoneal equilibration test
PF	peritoneal fibrosis
PSTR	peritoneal solute transport rate
ROS	reactive oxygen species
TGF-β	transforming growth factor-β
TLR	Toll-like receptor
TNF-α	tumor necrosis factor-α
UF	ultrafiltration
VEGF	vascular endothelial growth factor
vWF	von Willebrand factor
α-SMA	α-smooth muscle actin
